# Inflammatory Cytokine: An Attractive Target for Cancer Treatment

**DOI:** 10.3390/biomedicines10092116

**Published:** 2022-08-29

**Authors:** Hyang-Mi Lee, Hye-Jin Lee, Ji-Eun Chang

**Affiliations:** College of Pharmacy, Dongduk Women’s University, Seoul 02748, Korea

**Keywords:** inflammatory tumor microenvironment, inflammatory cytokine, cancer development, cancer prognosis, cancer treatment

## Abstract

The relationship between inflammation and cancer has attracted attention for a long time. The inflammatory tumor microenvironment consists of inflammatory cells, chemokines, cytokines, and signaling pathways. Among them, inflammatory cytokines play an especially pivotal role in cancer development, prognosis, and treatment. Interleukins, tumor necrosis factor-alpha (TNF-α), transforming growth factor-beta (TGF-β), interferons, and vascular endothelial growth factor (VEGF) are the representative inflammatory cytokines in various cancers, which may promote or inhibit cancer progression. The pro-inflammatory cytokines are associated with advanced cancer stages, resistance to immunotherapy, and poor prognoses, such as in objective response and disease control rates, and progression-free and overall survival. In this review, we selected colorectal, pancreatic, breast, gastric, lung, and prostate cancers, which are well-reported for an association between cancer and inflammatory cytokines. The related cytokines and their effects on each cancer’s development and prognosis were summarized. In addition, the treatment strategies targeting inflammatory cytokines in each carcinoma were also described here. By understanding the biological roles of cancer-related inflammatory cytokines, we may modulate the inflammatory tumor microenvironment for potential cancer treatment.

## 1. Introduction

Inflammation is an innate immune system that involves the recruitment and activation of immune cells, as well as the function of soluble factors, including cytokines and chemokines. This process has traditionally represented a front line of host defense against harmful stimuli, such as pathogens or irritants, and inflammatory cells are also essential for tissue repair [1]. Beyond this traditional role of inflammation, robust analyses of tumor transcriptomes indicated that inflammation is closely related to tumors by revealing a distinct expression profile of inflammatory cytokines and recruited immune cells in different tumor types [2,3]. In the context of ‘tumor-associated inflammation’, recent extensive studies have demonstrated a reciprocal interaction between inflammation and cancer, providing a comprehensive concept of tumor microenvironment (TME) where tumor cells exist in the network of stromal cells and cells of innate and adaptive immunity. While tumor cells regulate the inflammation state by secreting inflammatory mediators in TME, inflammation also controls cancer development, progression, and response to cancer therapies [4]. Tumor-cell-intrinsic changes during tumorigenesis can elicit inflammation. For instance, the loss of a tumor suppressor, e.g., p53, induces an increased activation of nuclear factor-κB (NF-κB) and an inhibition of DNA repair, which leads to the expression of inflammatory genes and the stimulation of the inflammation pathway [5,6]. Signaling through activated oncogenes can drive the generation of cytokines, chemokines, and various inflammatory factors [7]. In addition, tumor cells in chronic inflammation secrete cytokines (transforming growth factor (TGF)-β and interleukin (IL)-10) and chemokines to prevent dendritic cells (DCs) from the presentation of tumor antigens, and to recruit immunosuppressive cells, such as myeloid-derived suppressor cells (MDSCs), regulatory T cells, and M2 macrophages, thereby resulting in the generation of a cancer-promoting inflammation environment by suppressing anti-tumor immune responses [8]. On the other hand, the notion that inflammation plays a significant role in the regulation of cancer is currently well accepted. The immune system either promotes or suppresses all stages of cancer, as well as prognosis and the outcome of cancer treatment. Certain types of cancer are preceded by chronic inflammation, which occurs before tumor initiation and promotes cancer. As shown in colorectal or liver cancer cases, chronic inflammation, such as inflammatory bowel disease or chronic hepatitis, increases the risk of cancer [9]. Reactive oxygen species generated by macrophages during inflammation may cause the accumulation of mutations in normal tissues, and inflammatory cytokines can affect the pro-survival signaling, i.e., the STAT-3 activating pathway, of mutated cells [10,11]. In the course of tumor progression, inflammatory cytokines, including IL-6 and IL-17, facilitate the proliferation of tumor cells, and several cytokines can play an antagonizing role in the immune cells of anti-tumor responses. Inflammatory cytokines, including tumor necrosis factor-alpha (TNF-α) and IL-1b, can influence the expression of transcription factors that induce epithelial to mesenchymal transitions, which enable the dissemination of tumor cells [12]. However, acute inflammatory responses contribute to anti-tumor immunity in TME. Upon DCs uptaking tumor antigens and becoming mature, they induce inflammatory responses by regulating multiple immune cells, such as M1 macrophages and natural killer cells, via inflammatory cytokines, including interferon (IFN)-γ, IL-1, IL-12, and IL-15, etc. [13].

Here, we focus on the role of inflammatory cytokines in cancer and review the relationships of inflammatory cytokines with various cancer types. In this context, we discuss therapeutic approaches targeting inflammatory cytokines for the development of anti-cancer drugs. Given that the regulation of cancer is tightly engaged with inflammation, specifically inflammatory cytokines, defining the underlying mechanisms will provide insight into advanced strategies for the development of anti-cancer therapies.

## 2. Inflammatory Cytokines in Cancers

### 2.1. Colorectal Cancer

Colorectal cancer (CRC) refers to a malignant tumor composed of cancer cells in the large intestine. CRC is largely divided into colon or rectal cancer, depending on where cancer occurs. The incidence risk of CRC is associated with risk factors such as physical inactivity, age, race, or sex [14]. Chronic inflammation is considered to have a strong association with the early stages of tumor onset. CRC commonly occurs via a somatic mutation in a gene that encodes a part of the Wnt signaling pathway; hereditary mutations, such as nonpolyposis colorectal cancer (Lynch syndrome) [15]; or familial adenomatous polyposis [16]. Inherited cases can be prevented or delayed by anti-inflammatory treatment [17,18]. Inflammatory bowel diseases, including Crohn’s disease and ulcerative colitis, increase the risk of CRC related to colitis with poor prognoses [19,20]. Dietary and gut microbiota also affect the progression of chronic enteritis [21,22,23,24,25,26]. Gut microbial distribution changes as CRC progresses, and this change is related to pathological tumor characteristics [27,28]. While certain types of intestinal bacteria may protect the host by promoting an anti-inflammatory immune system, others can induce inflammation or mutation [21,22,23,24,25,26]. Since CRC is closely associated with chronic inflammation, various studies for inflammatory cytokines in CRC have been evaluated.

TNF-α is a well-known tumor-suppressive cytokine that induces apoptosis in specific types of cells. On the other hand, it promotes tumors so that inflammation can proceed to cancer [29,30,31]. Colitis and colitis-associated colon cancer (CAC) proceeded fast in a TNF-α–IL-10-deficient mouse model compared with an IL-10-deficient mouse model. In this study, TNF-α acted as a protective factor against inflammation and a tumor suppressor [32]. When TNF-α plays a tumor promoter role, a TNF-α inhibitor can be an attractive targeted treatment. In a study by Liu et al., a combination therapy of 5-fluorouracil (5-FU) and infliximab (TNF-α inhibitor) showed better outcomes than 5-FU monotherapy [33]. In more than 30% of CRC cases, granulocyte–macrophage colony-stimulating factor (GM-CSF) expression is high. GM-CSF is produced in the hematological part, which may increase anti-cancer immune responses. Overexpression of GM-CSF was strongly associated with increased overall survival rates of CRC patients [34]. Interestingly, when anti-programmed death-1 (PD-1) was used to treat a GM-CSF-silenced mice model, 25% tumor remission was found, while 50% tumor remission was observed from a GM-CSF-secreting mice model [34]. The combination of anti-PD-1 and GM-CSF showed synergetic anti-cancer effects. Another overexpressed inflammatory cytokine in CRC is IL-6. Inhibition of IL-6 or its receptors in a CAC-induced mouse model revealed a decreased tumor burden [11,35]. IL-1β also plays an important role in CRC oncogenesis with increased Toll-IL-1 receptor signaling [36,37]. Furthermore, the IL-1 receptor antagonist inhibited the metastatic process of CRC by suppressing the IL-1α/PI3K/NF-κB pathway [38]. A meta-analysis of serum IL-6 in CRC patients was performed, with a total of 17 studies. IL-6 is mainly produced by T cells, macrophages, and endothelial cells. Elevated serum IL-6 levels correlated with worse overall and disease-free survival rates for CRC [39]. Other inflammatory cytokines such as IL-8, IL-1 receptor antagonist (IL-1RA), and IL-6 were proven to be associated with advanced CRC [40]. The inflammatory cytokines were confirmed to be attractive biomarkers for CRC diagnosis and/or prognosis. Several clinical trials targeting inflammatory cytokines in CRC have been initiated. A phase I/II trial using antibody targeting IL-6 (siltuximab) [41], and a phase III trial of recombinant TNF receptor (etanercept) [42], failed to induce a clinical response. However, in metastatic CRC, MABp1 (IL-1α-targeted antibody) proved to be safe and effective in a phase I study [43]. The IL-1β inhibitor is known to increase the anti-tumor efficacy of 5-FU. In a phase II clinical trial using 5-FU, bevacizumab, and anakinra (IL-1β and α inhibitor) for patients with metastatic CRC, promising activity and a controllable safety profile were shown [44].

### 2.2. Pancreatic Cancer

Pancreatic cancer is one of the most disastrous cancers and shows a very poor prognosis. Current standards of care for pancreatic cancer are surgical resection with chemotherapy [45]. It shows the lowest 5-year survival rate among cancers between 2007 and 2013 [46]. In most pancreatic cancer cases, it is symptomless until it progresses, and this leads to a poor survival rate. Pancreatic cancer has some relevant risk factors, including cigarette smoking, diabetes mellitus, chronic pancreatitis, and obesity [47,48,49,50,51]. Recently, inflammation has been getting attention because it affects the development and progression of pancreatic cancer. The inflammation process is associated with some carcinogenic processes [52]. Several inflammatory cytokines are known to be related to the oncogenesis of pancreatic cancer.

IL-6 is a pro-inflammatory cytokine that shows diverse functions of cell multiplication, injury, infection, and inflammation [53]. It affects tumor cells to develop pancreatic cancer by controlling vascular endothelial growth factor (VEGF) secretion [54]. Targeting IL-6 was suggested to be one of the therapeutic approaches for pancreatic cancer [55]. IL-8 plays a key role in promoting the angiogenesis of pancreatic cancer. Primary sources for IL-8 are macrophages, platelets, and epithelial cells. IL-8 showed high levels in the serum of pancreatic cancer patients and in the human pancreatic cancer cell line [56,57]. The elevated IL-8 level was related to the low survival rate of pancreatic cancer patients, which has led it to be considered as a marker for prognosis [58]. Interestingly, serum levels of IL-6, IL-8, IL-10, and IL-1RA were significantly increased in pancreatic cancer patients. These cytokine levels were associated with worse survival rates, poor performance status, and/or weight loss [59]. TNF-α is associated with acute and chronic inflammation, autoimmune disease, and inflammation related to cancers [60]. It has two receptors: (i) TNF-receptor 1, which is distributed in all types of cells with a death domain that leads to apoptosis; (ii) TNF-receptor 2, which is only distributed in hematopoietic cells without a death domain. According to the study with a pancreatic-cancer-induced mouse model, TNF-α accelerated tumor growth and metastasis. Furthermore, anti-TNF-α treatment significantly inhibited tumor progression [61]. IL-1β is known to be related to inflammation responses [62], cancer progression [63], and cancer cell invasiveness [64] in pancreatic cancer. In this manner, IL-1β has attracted attention as another therapeutic target for pancreatic cancer. Macrophage migration inhibitory factor (MIF) appears to have a function as a pro-inflammatory cytokine that controls immune and inflammatory responses [65]. MIF is also known to be associated with tumor survival and progression [66,67]. From the phase I clinical study of imalumab (a fully human recombinant antioxidized MIF antibody), the maximum tolerated and biologically active doses have been investigated in pancreatic cancer patients [68]. TGF-β directly inhibits cell proliferation in pancreatic cancer and controls immune response [69]. In a phase I/II study, a TGF-β2-specific inhibitor was used as second-line therapy, and it showed significant improvements in clinical response compared with the current standard of care [70].

### 2.3. Breast Cancer

Breast cancer is a disease that makes the cells in the breast grow out of control. Breast cancer shows the highest incidence and cause of death in women [60]. It results in 14% of total cancer deaths worldwide [71]. Risk factors for breast cancer include age; genetic mutations, such as BRCA1 and BRCA2; reproductive history, and obesity. The initiation process of breast cancer is not clear; however, inflammation has been suggested as a cause for tumor initiation, progression, angiogenesis, and metastasis [72]. Inflammation is closely related the cancer, in that cell proliferation is mainly derived from inflammatory molecules.

TNF-α promotes the activation, differentiation, survival, or death of cancer cells under specific conditions. It also controls immune and inflammatory responses [73]. TNF-α is rarely detected in healthy women’s serum, while it exists in high levels in breast cancer patients [74,75]. The main cell sources for TNF-α are T cells and macrophages. When 93 breast carcinoma samples were analyzed, 97% of samples were positive for TNF-α. Among them, 61% were considered to be high-grade TNF-α. There was no correlation between TNF-α positivity and relapse-free or overall survival [76]. Anti-TNF-α treatment using a monoclonal antibody (infliximab) against a TNF receptor appears to repress tumor growth, induce tumor degeneration, and inhibit bone metastases in breast cancer-induced mice [77]. TGF-β1 is considered as a prognosis marker for breast cancer. It is mainly produced by T cells and macrophages. Breast cancer patients with high TGF-β1 plasma levels had significantly worse overall and disease-free survival rates [78]. Elevated TGF-β1 levels in metastatic axillary lymph node tissue were associated with metastatic axillary lymph node numbers and tumor size [79]. In breast cancer mouse models, blocking TGF-β signaling was effective in decreasing tumor growth and metastasis [80]. IL-6 was suggested to be another prognostic biomarker of breast cancer. In a study with 87 patients who had hormone-refractory metastatic breast cancer, high levels of IL-6 were notably related to poor survival [81]. IL-12 controls the immunity and inflammatory reactions that mediate cancer progression. It has pro-inflammatory functions via activating cytotoxic immune cells [82]. A phase II clinical study (NCT04095689) using chemotherapy and pembrolizumab plus IL-12 gene therapy with triple-negative breast cancer is ongoing. The combination of chemotherapy and pembrolizumab was proven to enhance the anti-tumor efficacy. In addition, IL-12 gene therapy stimulates the anti-tumor immune response [83]. Gene therapy based on GM-CSF has been proven for its efficacy and safety through clinical trials. In the phase I study, various cancers, including breast cancer, were treated with oncolytic herpes simplex virus expressing GM-CSF. The anti-tumor immune response and tumor necrosis were observed as having a safe profile [84].

### 2.4. Gastric Cancer

The incidence and mortality rates of gastric cancer have been constantly declining. However, it is still the fifth most common cancer and the fourth leading cause of deaths related to cancer [71]. Among the many factors influencing gastric cancer, chronic atrophic gastritis is most closely related to the occurrence of gastric cancer [85]. Gastric inflammation is commonly caused by Helicobacter pylori and autoimmune gastritis. Gastric inflammation leads to atrophic gastritis, metaplasia, dysplasia, and adenocarcinoma [86,87]. In addition, chronic gastric inflammation increases the risk of gastric cancer. Various cytokines secreted from immune and epithelial cells in chronic inflammation are identified, and they are expected to be potential targets for gastric cancer treatment.

In a clinical study with gastric ulcer patients, IL-17 was proved to be important in the inflammatory response to Helicobacter pylori. Moreover, IL-17 also affects the Helicobacter pylori-associated diseases. IFN-γ showed increased levels in gastric mucosa after Helicobacter pylori infection. IFN-γ upregulates NF- κB signaling so that carcinogenesis occurs [88]. Accordingly, inhibition of IFN-γ can be a key treatment for gastric cancer. IL-6 is a pro-inflammatory cytokine that promotes the growth and progression of gastric cancer. It was identified that IL-6 is overexpressed in the stromal portion of gastric cancer and the elevated IL-6 stimulates the Jak1-STAT3 pathway in gastric cancer via paracrine signaling. This leads to the development of stroma-induced chemoresistance. To overcome the resistance to chemotherapy by targeting IL-6, tocilizumab (anti-IL-6 receptor monoclonal antibody) was used in treatment and it effectively enhanced the anti-tumor effect of chemotherapy in gastric cancer [89]. Several inflammatory cytokines were evaluated to determine whether they may be applied as prognostic biomarkers. Gastric cancer patients with high-IL-17-serum concentrations showed significantly lower 5-year survival rates compared with patients with low IL-17 rates [90]. The expression of IL-22 receptors in gastric cancer appears to be associated with lymphatic invasion and poor prognosis [91]. Furthermore, high levels of IL-6 were also related to poor prognosis with recurrence and the overall survival rates of gastric cancer patients [92]. In a clinical trial, gene therapy using GM-CSF has been proven useful for its efficacy and safety against gastric cancer [84]. Currently, PD-1/programmed death ligand-1 (PD-L1) immune checkpoint inhibitors (ICIs) are often selected for cancer treatment. ICIs inhibit the immunosuppressive mechanisms of tumor cells. ICIs utilize host autoimmune functions for antitumor activity while anti-cancer agents attack the cancer cells directly. However, unfortunately, only a few selected cancer patients responded to this immunotherapy due to different PD-1/PD-L1 expression levels. Infiltrated macrophage and PD-L1 expression in gastric cancer showed high correlation. IL-6 and TNF-α from macrophages induce PD-L1 via the NF-κB and STAT3 signaling pathways. Elevated PD-L1 levels in gastric cancer cells promote the proliferation of gastric cancer cells [93]. IL-6, TNF-α, and PD-L1 may be attractive targets for gastric cancer treatment.

### 2.5. Lung Cancer

Lung cancer remains a major public health issue worldwide with both high incidence and mortality rates [71]. Most lung cancers do not cause any typical symptoms, such as cough, chest pain, shortness of breath, and hoarseness, at an early stage. Delayed diagnosis may lead to poor survival rates in lung cancer patients [94]. According to the American Cancer Society’s report, the 5-year relative survival rates of patients with a distant metastasis of lung cancer are 8% and 3% for non-small cell lung cancer (NSCLC) and small-cell lung cancer (SCLC), respectively [14]. Understanding the specific inflammatory cytokines from the lung cancer will help for early diagnosis and novel treatment strategies.

From previous pre-clinical studies, the correlation between inflammatory cytokines and lung cancer progression has been suggested. TNF-α acts as either a tumor suppressor or promoter in lung cancer. As a tumor suppressor, TNF-α played an essential role in CD8 T cell-mediated lung cancer cell elimination in vivo. The tumor regression from a lung cancer mouse model using CD8 T cell epitope was dependent on TNF-α levels [95]. On the other hand, as a tumor promoter, TNF-α attenuated Fas-induced A549 human lung carcinoma cell death. This anti-cancer cell death effect was affected by NF-κB, phoshatidylinositole-3 kinase (PI3-K), and mitogen-activated protein kinase (MAPK) pathways, in addition to increased anti-apoptotic protein and FLICE-like inhibitory protein long (FLIPL) [96]. In addition, TNF-α was proved to stimulate tumor growth and metastasis in lung cancer-bearing mice. The tumor promoting effect of TNF-α was dependent on the activity of NF-κB, which induces anti-apoptotic proteins, such as B-cell lymphoma-extra large (Bcl-XL), cellular inhibitor of apoptosis protein (cIAP)1, and cIAP2 [97]. In these cases, the anti-TNF-α drugs were suggested to reduce inflammation-induced tumor progression. IL-4 receptors were also expressed in lung cancer, and when the IL-4 receptor-targeted agent was administered, high cytotoxicity was observed in lung cancer cells and lung cancer-induced mice. IL-4 cytotoxin was proposed as a novel therapeutic approach for lung cancer [98]. VEGF is another overexpressed inflammatory cytokine in lung cancer. A combination of the epidermal growth factor receptor (EGFR) tyrosine kinase inhibitor (TKI) and anti-VEGF enhanced the anti-tumor efficacy to overcome the EGFR TKI resistance in lung cancer-induced mice [99]. The VEGF inhibitors bevacizumab and ramucirumab have been approved for NSCLC treatment in combination with chemotherapy [100]. Evidence from clinical studies also proved the correlation between inflammatory cytokines and lung cancer prognosis. Song et al. enrolled 48 NSCLC patients and 40 healthy controls to evaluate the relationship between inflammatory cytokines and NSCLC. The levels of TNF-α, IL-6, IL-8, and VEGF in serum were significantly higher in NSCLC patients compared with the healthy control group. Furthermore, TNF-α, IL-8, and VEGF levels were increased in accordance with the advanced stages of NSCLC [101]. Brenner’s team conducted a case–control study with 807 lung cancer patients and 807 smoking-matched controls. Lung cancer patients showed increased IL-6 and IL-8 levels in the serum, and the association was stronger among former and current smokers. In the study, it was also found that IL-6 and IL-8 levels are increased many years before the diagnosis of lung cancer [102]. From another clinical study, IL-10 and TGF-β1 from NSCLC patients showed significantly higher serum levels than both the healthy control and benign tumor groups. Down-regulated DNA methyltransferases increased forkhead box protein P3 (Foxp3) genes, and consequently these Foxp3+ T cells induced the immunosuppressive cytokines IL-10 and TGF-β1. The immunosuppressive microenvironment led to the tumor progression of NSCLC [103]. Inflammatory cytokines were also applied to predict the ICI treatment response from NSCLC patients. IL-10 and IL-10 receptors were found in lung cancer tissue from NSCLC patients and lung cancer cell line cultures. A positive correlation was discovered between IL-10 level and tumor size, which resulted in poor prognosis. IL-10 counteracted IFN-γ effects on the PD-1/PD-L1 pathway, which induced tumor resistance to ICIs. In this manner, IL-10 may be used to predict ICI treatment success forecasts in NSCLC before ICI treatment. Patients with lower IL-10 will show better ICI response and prognosis [104]. From another recent study, 125 NSCLC patients who received a PD-1/PD-L1 inhibitor were enrolled to investigate whether baseline serum IL-6 levels may predict ICI treatment efficacy. The subjects with low IL-6 (<13.1 pg/mL) showed significantly higher objective response and disease control rates than those with high IL-6 levels. In addition, the median progression-free and overall survival rates were significantly longer in the low-IL-6 group compared with the high-IL-6 group. In this study, the IL-6 levels in serum were suggested as a potential biomarker to predict the efficacy of ICI treatment in NSCLC [105].

### 2.6. Prostate Cancer

Prostate cancer is one of the most challenging cancer types among men. Cancer progression and therapeutic resistance often lead to high mortality rates [106]. Prostate cancer ranks as the second leading cause of cancer-related deaths among American men [107]. Well-known risk factors for prostate cancer include older age [108], black race [109,110], BRCA mutations [111,112], and family history [113]. Interest in the linkages between inflammation and prostate cancer has increased. Chronic inflammatory disease, such as prostatitis, was proved to increase the risk of prostate cancer [114,115]. On the other hand, some negative associations between chronic inflammation and prostate cancer have also been reported [116,117]. Knowledge of the role of inflammatory cytokines in prostate cancer progression may provide an optimized targeted-therapy strategy.

IL-30 overexpression by prostate cancer stem-like cells (PCSLC) promoted tumor onset and progression in vivo. IL-30 also played a critical role in PCSLC spread to lymph nodes and bone marrow by increasing the CXCR5/CXCL13 axis and lung metastasis through the CXCR4/CXCL12 axis. In this study, suppressing PCSLC by the proper targeting of upstream drivers was suggested as a potential treatment against prostate cancer progression and recurrence [118]. Interestingly, TGF-β1 shows double-faced functions in prostate cancer progression. At early stages, it acts as a cancer growth inhibitor, while at advanced stages, it promotes cancer development [119,120]. Park et al. reported that TGF-β1 activates IL-6 in human prostate cancer cells via synergistic signaling pathways, which are Smad2, p38-NF-κB, JNK, and Ras. IL-6 accelerates cancer cell proliferation and survival, which influence the progression and metastasis of prostate cancer. In addition, elevated IL-6 may contribute to the conversion of TGF-β1′s role as a prostate cancer promoter. Anti-IL-6 neutralizing antibody or antisense IL-6 effectively inactivated IL-6 signaling, leading to TGF-β-mediated apoptosis [121]. Various clinical trials also showed the association between inflammatory cytokines and prostate cancer prognosis. First, IL-6 levels in serum are increased in patients with prostate cancer, and it significantly correlated with cancer prognosis. A clinical study from Nakashima’s team measured IL-6 levels in serum samples from stages B, C, and D prostate cancer patients. In this study, high serum IL-6 levels were associated with the advanced stages of prostate cancer and poor survival rate [122]. IL-6 has been reported to increase erythrocyte sedimentation rate [123], which was proved to be a prognostic factor in the survival of advanced prostate cancer patients [124]. Another study from Michalaki et al. reported elevated IL-6 and TNF-α serum levels in prostate cancer patients compared with healthy controls. These increased inflammatory cytokines were correlated with advanced stages, metastasis, and poor overall survival in patients with prostate cancer [125]. TNF-α was also suggested to play an important role in the development of cachexia from prostate cancer patients. The patients with high-TNF-α serum levels showed higher performance status and mortality rates than the patients with undetectable TNF-α serum levels [126]. IL-17 is another inflammatory cytokine overexpressed in prostate cancer. Steiner et al. performed a screening of inflammatory cytokines from normal, benign hyperplastic, and malignant prostate tissues. IL-17 was rarely expressed in the normal prostate, whereas its expression was increased in benign hyperplastic and malignant prostates. In addition, a significant correlation was monitored between IL-17 level and both IL-6 and IL-8 levels in malignant prostate specimens [127].

## 3. Conclusions

**Figure 1:**
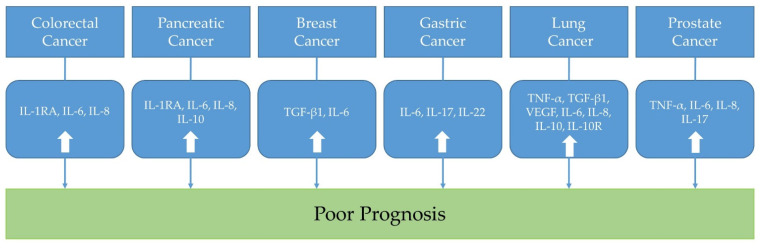
The association between inflammatory cytokines in cancers and prognosis. (IL: interleukin; IL-1RA: IL-1 receptor antagonist; TNF-α: tumor necrosis factor-α; TGF: transforming growth factor; VEGF: vascular endothelial growth factor; IL-10R: interleukin-10 receptor).

In this review, representative inflammatory cytokines in colorectal, pancreatic, breast, gastric, lung, and prostate cancers were discussed. From the preclinical studies using cancer cell lines and cancer-induced animal models, some potential treatment strategies targeting inflammatory cytokines were suggested (Table 1). In addition, from the clinical trials, the associations between inflammatory cytokines and cancer prognosis were evaluated (Table 2 and Figure 1). Taken all together, by understanding the biological roles of inflammatory cytokines toward the cancers, we may modulate the inflammatory tumor microenvironment to find ideal cancer-targeted treatment.

## Figures and Tables

**Table 1:** The suggested treatment strategies from preclinical studies according to inflammatory cytokines in cancers.

Cancer Type	Inflammatory Cytokine	Treatment Strategy	Reference
Colorectal	TNF-α	TNF-α inhibitor	[29,30,31,33]
	GM-CSF	combination of anti-PD-1 and GM-CSF	[34]
	IL-6	inhibition of IL-6 or its receptor	[11,35]
	IL-1β	IL-1β inhibitor	[36,37]
	IL-1	IL-1 receptor antagonist	[38]
Pancreatic	IL-6	IL-6 signaling inhibitor	[55]
	IL-8	anti-IL-8 neutralizing antibody	[56,57]
	TNF-α	TNF-α inhibitor	[61]
Breast	TNF-α	anti-TNF-α monoclonal antibody	[77]
	TGF-β	blocking TGF-β signaling	[80]
Gastric	IFN-γ	IFN-γ inhibitor	[88]
	IL-6	anti-IL-6 receptor monoclonal antibody	[89]
Lung	TNF-α	TNF-α inhibitor	[96,97]
	IL-4R	IL-4 cytotoxin	[98]
	VEGF	combination of VEGF inhibitor and EGFR TKI	[99]
Prostate	IL-30	suppressing PCSLC	[118]
	TGF-β1 and IL-6	anti-IL-6 neutralizing antibody or antisense IL-6	[121]

TNF: tumor necrosis factor; GM-CSF: granulocyte–macrophage colony-stimulating factor; PD-1: programmed death-1; IL: interleukin; TGF: transforming growth factor; IL-4R: interleukin-4 receptor; VEGF: vascular endothelial growth factor; EGFR: epidermal growth factor receptor; TKI: tyrosine kinase inhibitor; PCSLC: prostate cancer stem-like cells.

**Table 2:** The association between inflammatory cytokines in cancers and prognosis from clinical studies.

Cancer Type	Inflammatory Cytokine	Source	Prognosis	Reference
Colorectal	GM-CSF	tissue	high GM-CSF level had better OS	[34]
	IL-6	serum	high IL-6 level had poor OS and DFS	[39]
	IL-8, IL-1RA and IL-6	serum	high IL-8, IL-1RA and IL-6 levels had advanced stages	[40]
Pancreatic	IL-8	serum	IL-8 positive had poor survival	[58]
	IL-6 and IL-10	serum	high IL-6 and IL-10 levels had poor survival	[59]
	IL-1RA	serum	low IL-1RA level had poor survival	
	IL-6, IL-10 and IL-8	serum	high IL-6, IL-10 and IL-8 levels had poor performance status and/or weight loss	
Breast	TNF-α	tissue	no correlation between TNF-α positivity and RFS or OS	[76]
	TGF-β1	serum	high TGF-β1 level had poor OS and DFS	[78]
		tissue	high TGF-β1 level had more metastatic axillary lymph nodes and increased tumor size	[79]
	IL-6	serum	high IL-6 level had poor survival	[81]
Gastric	IL-17	serum	high IL-17 level had poor 5-year survival	[90]
	IL-22	tissue	high IL-22 receptors had more lymphatic invasion and poor prognosis	[91]
	IL-6	serum	high IL-6 level had poor recurrence and OS	[92]
Lung	TNF-α, IL-6, IL-8 and VEGF	serum	high TNF-α, IL-8 and VEGF levels had advanced stages	[101]
	IL-6 and IL-8	serum	high IL-6 and IL-8 had high lung cancer risk	[102]
	IL-10 and TGF-β1	serum	high IL-10 and TGF-β1 levels had high lung cancer risk	[103]
	IL-10 and IL-10R	tissue	high IL-10 level had increased tumor size, resistance to ICI and poor prognosis	[104]
	IL-6	serum	low IL-6 level had increased ORR, DCR, PFS and OS in ICI treated patients	[105]
Prostate	IL-6	serum	high IL-6 level had advanced stages and poor survival	[122]
	IL-6 and TNF-α	serum	high IL-6 and TNF-α levels had advanced stages, metastasis and poor OS	[125]
	TNF-α	serum	high TNF-α level had poor performance status, poor mortality rate and cachexia development	[126]
	IL-17, IL-6 and IL-8	tissue	high IL-17, IL-6 and IL-8 levels had advanced stages	[127]

GM-CSF: granulocyte–macrophage colony-stimulating factor; IL: interleukin; IL-1RA: IL-1 receptor antagonist; TNF-α: tumor necrosis factor-α; MIF: macrophage migration inhibitory factor; TGF: transforming growth factor; VEGF: vascular endothelial growth factor; IL-10R: interleukin-10 receptor; ICI: immune checkpoint inhibitor; DFS: disease-free survival; RFS: relapse-free survival; ORR: objective response rate; DCR: disease control rate; PFS: progression-free survival; OS: overall survival.

## Abbreviations

TME	tumor microenvironment
NF-κB	nuclear factor-κB
TGF	transforming growth factor
IL	interleukin
DC	dendritic cell
MDSC	myeloid-derived suppressor cell
TNF	tumor necrosis factor
IFN	interferon
CRC	colorectal cancer
CAC	colitis-associated colon cancer
5-FU	5-fluorouracil
GM-CSF	granulocyte–macrophage colony-stimulating factor
IL-1RA	IL-1 receptor antagonist
VEGF	vascular endothelial growth factor
MIF	migration-inhibitory factor
PD-1	programmed death-1
PD-L1	programmed death ligand-1
ICI	immune checkpoint inhibitor
NSCLC	non-small-cell lung cancer
SCLC	small-cell lung cancer
PI3-K	phoshatidylinositole-3 kinase
MAPK	mitogen-activated protein kinase
FLIPL	FLICE-like inhibitory protein long
Bcl-XL	B-cell lymphoma-extra large
cIAP	cellular inhibitor of apoptosis protein
EGFR	epidermal growth factor receptor
TKI	tyrosine kinase inhibitor
Foxp3	forkhead box protein P3
PCSLC	prostate cancer stem-like cells
